# Complete plastid genome sequence of *Neolitsea aciculata* (Laurales: Lauraceae), an evergreen broad-leaved tree endemic to East Asia

**DOI:** 10.1080/23802359.2021.1959457

**Published:** 2021-08-02

**Authors:** Eun-Kyeong Han, Gantsetseg Amarsanaa, Jung-Hyun Kim, Soonku So, In-Su Choi, Jung-Hyun Lee

**Affiliations:** a Department of Biological Sciences and Biotechnology, Chonnam National University, Gwangju, Republic of Korea; b Department of Biology Education, Chonnam National University, Gwangju, Republic of Korea; c Plant Resources Division, National Institute of Biological Resources, Incheon, Republic of Korea; d Ecosystem Research Division, Korea National Park Research Institute, Wonju, Republic of Korea; e School of Life Sciences, Arizona State University, Tempe, AZ, USA

**Keywords:** Chloroplast, Lauraceae, *Neolitsea aciculata*, phylogenetic analysis, pseudogene

## Abstract

In this study, we sequenced the complete plastid genome (plastome) of *Neolitsea aciculata*, an evergreen broad-leaved tree endemic to East Asia, a woody component of East Asian warm-temperate and subtropical forests across China, Korea, and Japan. The plastome of *N. aciculata* is assembled as a single contig (152,722 bp). A large and a small single copy (93,785 and 18,795 bp, respectively) of the genome are separated by a pair of inverted repeats (20,071 bp). The genome consists of 126 genes, including 80 protein-coding, eight ribosomal RNA, and 36 transfer RNA genes. Two genes in the IR region (*ycf*1 and *ycf*2) are pseudogenized. Our phylogenetic analysis revealed the phylogenetic position of *N. aciculata* in a highly supported clade of the genus *Neolitsea* along with other two congeners, *N. pallens* and *N. sericea.*

The family Lauraceae consists of approximately 50 genera and 2,500–3,000 species worldwide (Chanderbali et al. 2001). *Neolitsea* (Benth.) Merr., a major genus of the family comprises ca. 100 species widely distributed throughout the tropical and subtropical habitats in Asia, including China, Korea, and Japan (Gentry 1988; van der Werff 2001; Lee 2003; Ohba 2006; Huang and van der Werff 2008). Its high species diversity is of considerable evolutionary importance as a core genus of Laureae, *Litsea* complex (Li et al. 2004, 2007). However, their phylogenetic relationships are still poorly understood.

To date, only two *Neolitsea* plastid genomes (plastomes) have been sequenced. A study on the woody plants, *N. pallens* (D. Don) Momiyama and H. Hara and *N. sericea* (Blume) Koidz., has provided powerful phylogenetic utilities, regarding the *Litsea* complex in particular (Xiao et al. 2020). However, given their high species diversity, such deficiency in the plastome sequence resources on the genus level is a hindrance to improving our understanding of the woody plant-related evolutionary processes in tropical and subtropical forests. *Neolitsea aciculata* (Blume) Koidz. 1918 is a woody plant of East Asian warm-temperate and subtropical forests across China, Korea, and Japan. In this study, we sequenced and characterized a complete plastome from *N. aciculata*, endemic to East Asia.

*N. aciculata* samples were collected from the Jeju Island, South Korea (33°18′26.7″N, 126°27′09.9″E). The voucher specimen (Lee-Na200529) was stored in the department of Biology Education, Chonnam National University (BEC: quercus@jnu.ac.kr). The DNA library was constructed and sequenced using an MGI-seq 2000 platform (LAS, Seoul, Republic of Korea) following the manufacturer’s protocol. It generated 75,078,260 raw reads (150 bp paired-end). The *N. aciculata* plastome was assembled using NOVOPlasty 4.1 (Dierckxsens et al. 2017), using the *N. pallens mat*K gene sequence (Xiao et al. 2020; MN428466) as the seed. The assembled plastome was verified using Geneious 11.0.5 (Kearse et al. 2012) by reference mapping 381,661 reads, resulting in a coverage of 150×. The annotation was separately performed using Geneious 11.0.5 and manually corrected for the start and stop codons, as well as the intron-exon boundaries. The annotated plastome sequence was deposited in the GenBank (accession number: MW845678). To construct the phylogenetic tree, plastomes of 10 Lauraceae species (*Neolitsea*, *Lindera,* and *Litsea*: two species each, and *Cinnamomum, Laurus*, *Actinodaphne*, and *Machilus*: one species each, respectively) were downloaded from the NCBI database. The alignments were performed using MAFFT (Katoh and Toh 2010). The maximum likelihood (ML) analysis was performed with RAxML v.8.0 (Stamatakis 2014) using default parameters and 1000 bootstrap replicates. For the RAxML tree, the general time-reversible (GTR) model of nucleotide substation was used with the Gamma model of rate heterogeneity.

Our results showed that the *N. aciculata* plastome is 152,722 bp long, with two inverted repeat (IR) regions (20,071 bp each) that separate a large single copy (LSC) region (93,785 bp) and a small single copy (SSC) region (18,795 bp). It contains 126 genes, including 80 protein-coding, eight ribosomal RNA, and 36 transfer RNA genes. Two genes in the IR region, *ycf*1 and *ycf*2, are pseudogenized as the nucleotide sequences of the 1,383 bp of 3′–*ycf*1 and 3,162 bp of 5′–*ycf*2 truncated at the IR boundaries. The *N. aciculata* plastome is similar in its gene content and order to those of two congeners, *N. pallens* and *N. sericea*. The pseudogenized *ycf*1 and *ycf*2 are also present in both congeners. The G + C content is overall 39.1%, of which the LSC, the SSC, and IR regions account for 37.9%, 33.9%, and 44.4%, respectively. Thirteen complete genes (three protein-coding, six tRNA, and four rRNA genes) were duplicated in the IR regions. Fifteen genes exhibit a single intron (*rps*16, *atp*F, *rpo*C1, *pet*B, *pet*D, *rpl*16, *rpl*2, *ndh*B, *ndh*A, *trn*K-UUU, *trn*G-UUC, *trn*L-UAA, *trn*V-UAC, *trn*I-GAU, and *trn*A-UGC) and three genes have two introns (*ycf*3, *clp*P, and *rps*12).

The ML phylogenetic tree shows that *N. aciculata* is closely related to *N. pallens* and *N. sericea* with strong (100%) bootstrap support. The genus *Neolitsea* was distinguished from the *Lindera*-related *Litsea* complex, forming a sister clade with *Actinodaphne obovata* (Figure 1). The complete plastome sequenced in this study represents valuable genomic resource data of the Lauraceae, and this new phylogenetic information could be used in future evolutionary studies of the Lauraceae.

**Figure 1.**
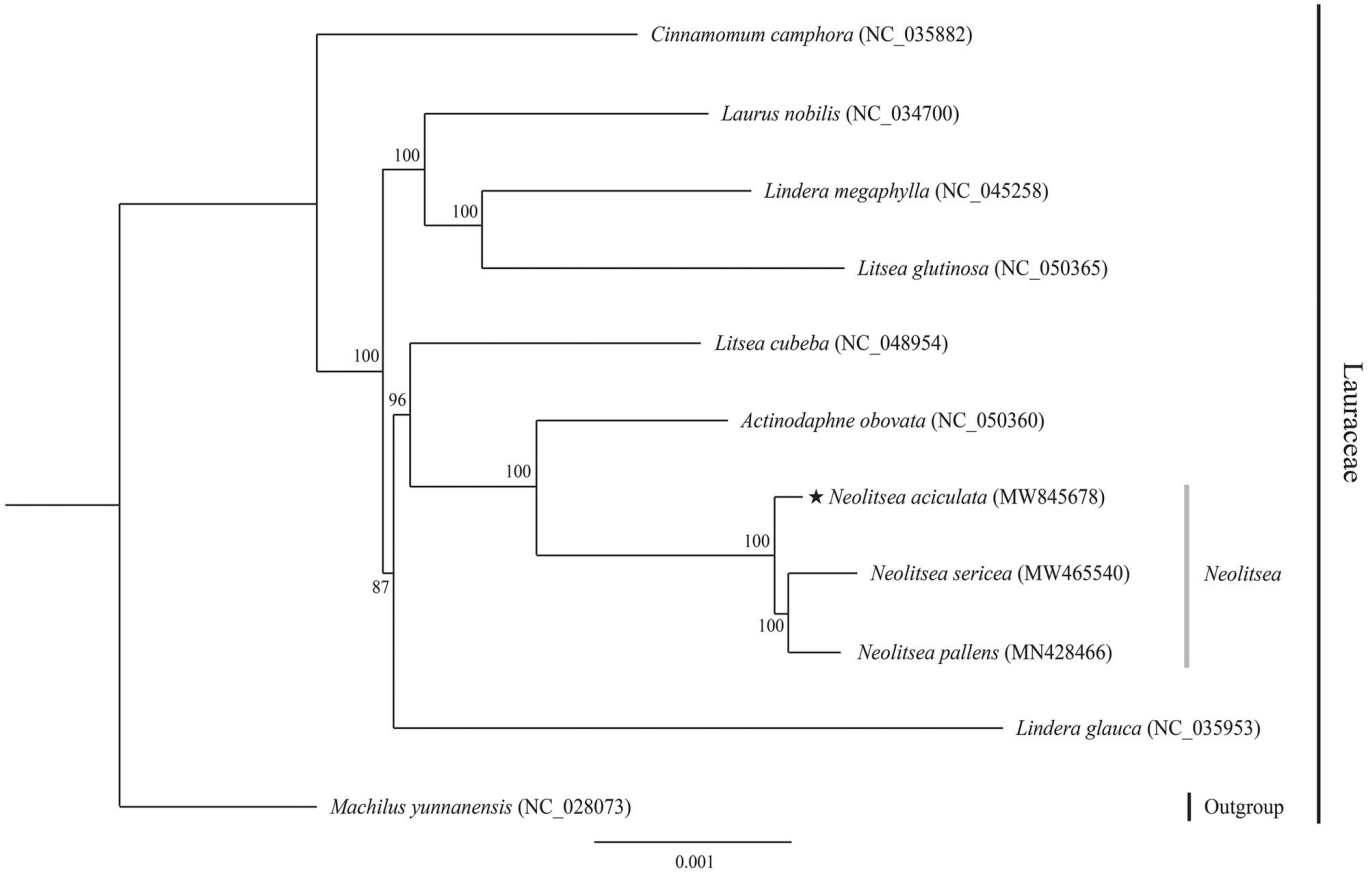
Phylogenetic tree (RAxML) established based on the plastome sequences of 11 species of Lauraceae and 71 common genes from 11 complete chloroplast genomes. *Machilus yunnanensis* was used as an outgroup. The numbers above the nodes indicate bootstrap values with 1000 replicates.

## Data Availability

The genome sequence data that support the findings of this study are openly available in the NCBI GenBank at [https://www.ncbi.nlm.nih.gov] (https://www.ncbi.nlm.nih.gov/) under the accession no. MW845678. The associated BioProject, SRA, and Bio-Sample numbers are PRJNA726624, SRS8815750, and SAMN18948665, respectively.
